# Antioxidant, antiapoptotic and amino acid balance regulating activities of 1,7-dihydroxy-3,4,8-trimethoxyxanthone against dimethylnitrosamine-induced liver fibrosis

**DOI:** 10.1371/journal.pone.0189344

**Published:** 2017-12-12

**Authors:** Xi-Yuan Zheng, Xin Zhao, Ying-Fan Yang, Han-Jie Jiang, Wan Li, Yi Sun, Xiao-Ping Pu

**Affiliations:** 1 National Key Research Laboratory of Natural and Biomimetic Drugs, Peking University, Beijing, P. R. China; 2 Department of Molecular and Cellular Pharmacology, School of Pharmaceutical Sciences, Peking University, Beijing, P. R. China; IDIBAPS Biomedical Research Institute, SPAIN

## Abstract

Liver fibrosis represents the consequences of a sustained wound healing response to chronic liver injury which could be caused by viral, autoimmune, drugs, and so on. Unfortunately, there was no effective therapy available for liver fibrosis in clinic. In this study, we identified the anti-fibrotic effects of 1,7-dihydroxy-3,4,8-trimethoxyxanthone (ZYC-1) on the dimethylnitrosamine (DMN)-induced rat model. ZYC-1 was isolated from *Swertia punicea* Hemsl and was administrated to DMN-induced rat model. ZYC decreased the hyaluronic acid (HA), type IV collagen (CIV) and hydroxyproline (Hyp) levels and inhibited the expression of α smooth muscle actin (α-SMA) and transforming growth factor beta 1 (TGF-1β). The anti-fibrotic effect of ZYC-1 was also confirmed by Sirius Red staining. Finally, we identified 42 differentially expressed proteins by using proteomics analysis after ZYC-1 treatment, of which 17 were up-regulated and 25 were down-regulated. These Most of the 42 proteins are involved in the oxidative stress pathway, the mitochondrial-mediated apoptotic pathway and the amino acid metabolism pathway. Our study presented the first elucidated mechanisms of xanthone on liver fibrosis *in vivo*. This study pointed out that ZYC-1 may be used as a lead compound for hepatofibrosis treatment.

## Introduction

It is estimated that 350 million people or so worldwide and 120 million in China alone are suffering from chronic liver injury [1]. Liver fibrosis represents the results of a sustained wound healing response to chronic liver injury from a variety of causes, including viral, autoimmune, drugs, cholestatic, alcoholic, and metabolic diseases [2]. Prior studies have indicated that liver fibrosis is potentially reversible, meaning the early diagnosis and intervention of this disease might have important clinical implications [3]. Dimethylnitrosamine (DMN) is a hepatotoxin that is well-known to cause acute liver injury in rats and reproduce the features of human liver fibrosis and cirrhosis, together with collagen accumulation, hepatocyte apoptosis, oxidative stress elevation and lipid peroxidation [4]. Since liver fibrosis has been showed to be a reversible process, efforts should be made in order to explore new therapeutic approaches. Unfortunately, no established anti-fibrotic therapies are currently available [5].

Traditional Chinese medicine (TCM), owing to its good efficacy and few side effects, has displayed unique features in regard to the treatment of liver diseases for thousands of years. Varieties of herbal extracts and compounds have been demonstrated to have anti-fibrotic properties [6, 7]. *Swertia punicea* Hemsl. (Gentianaceae) is a Traditional Chinese Medicine with numerous therapeutic applications, such as in the treatment of acute bilious hepatitis and cholecystitis [8]. Our group has preliminarily confirmed the hepatoprotective effects of 1, 7-dihydroxy-3, 4, 8-trimethoxyxanthone (ZYC-1), a xanthone compound extracted from *Swertia punicea*, both *in vivo* and *in vitro* [9]. However, the hepatoprotective mechanisms of ZYC-1 have not been fully understood yet.

In the present study, we further verified the hepatoprotective effect of ZYC-1 and determined hepatoprotective and the anti-fibrotic mechanisms of ZYC-1 on oxidative stress, mitochondrial-mediated apoptosis and amino acid imbalance based on the results of proteomics analysis in DMN-induced rat liver fibrosis model. Our study presented the first elucidated mechanisms of xanthone on liver fibrosis *in vivo*.

## Materials and methods

### Animals

All male SD rats weighing 180–200 g were purchased from the Department of Laboratory Animal Science of Peking University Health Science Center (Beijing, China). The rats were maintained in a specific pathogen-free facility with a 12h/12h cycle and received humane treatment. All animal-related procedures were approved by the Animal Care and Use committee of Peking University Health Science Center with confirmation number: LA2017107.

### Liver fibrosis animal model

Experimental Rats were randomly divided into 4 groups: normal (n = 10), model (n = 10), IFN-α2b Treatment (n = 10), and ZYC-1 treatment (n = 4). The liver interstitial fibrosis model was induced by i.p. injection of dimethylnitrosamine (DMN, Tokyo Kasei Kogyo Co., Ltd., Tokyo, Japan) with a dose of 10 mg·kg^-1^ body weight once every other day for 4 weeks [10]. Starting from the third week of DMN injection, rats in the treatment groups were treated with Interferon α2b at a dose of 8×10^5^ IU·kg^-1^ body weight or ZYC-1 at a dose of 5 mg·kg^-1^ body weight, respectively, once a day for 4 weeks. Rats in normal and model groups were dosed with equal amount of vehicle. At the end of the treatment, venous blood was withdrawn from each rat in the orbit under ether anesthesia, before the rat was sacrificed and perfused with saline so that the blood could be washed out, and then their livers and spleen were extracted. The flowchart of animal experiment was shown in the supporting information (S2 Fig).

### Liver fibrosis indices in rat sera

Hyaluronic acid (HA) and type IV collagen (CIV) were detected using the radioimmunoassay method by HA or CIV radioimmunoassay kit (Beijing North Institute of Biological Technology, Beijing, China). The experiments conducted in accordance with the manufacturer’s instructions.

As for the hydroxyproline (Hyp) level, 100 mg of liver tissue was homogenized and dissolved in 1 mL of 6 mol/L HCL at 95°C for 5 h. And then PH value of hydrolysate is adjusted to 6.0 ~ 6.8, and 3 ~ 4ml diluted hydrolysate was removed to a clean tube, and added certain amount of activated carbon (about 20 ~ 30mg) and mixed, and then separated by centrifugation at 3000rpm for 10 min at 4°C. The supernatant was measured using commercially available assay kits (Jiancheng, Nanjing, China) in accordance with the manufacturer’s instructions.

### Histological analyses

The dissected livers were fixed in formalin and embedded in paraffin. The sections were stained with Picro Sirius red [11]. To assess hepatocyte activation, the sections were further processed for immunohistochemistry (IHC) with TGF-β1 antibody(sc-146, 1:50 dilution) and α-SMA antibody(ab5694, 1:100 dilution). Sections were stained using the immunoperoxidase technique and then counterstained with haematoxylin [12].

### Proteomics

100 mg of liver tissue was lysed in lysis buffer consisting of 150 mM NaCl, 80 mM Tris-HCl (pH 7.4), 2 mM EDTA-2Na, 0.1% sodium dodecyl sulfate (SDS), 1 mM DTT and protease inhibitor cocktail. After insoluble fractions were removed by centrifugation at 12,000 g (4°C) for 20 min, the supernatant was collected and the protein concentrations were determined with a Bradford protein assay kit [13]. A 12.5% polyacrylamide gel was prepared for SDS-PAGE analysis. The 3 samples containing 200 μg proteins from the 3 rats of the respective group were mixed, boiled for five minutes, and then loaded onto the polyacrylamide gel. The gel was run for 30 minutes at 50 V and then 120 minutes at 100 V, and stained using Coomassie Blue R-250 for proteomics analysis.

Protein samples were cut from the gel in the range 12.5 kDa to 60 kDa. Each lane was top-down average divided into 8 bands of equal size. Each band was digested in-solution using 0.1 μg of trypsin in 25 mM ammonium bicarbonate solution in a total volume of 50 μl. After overnight incubation at 37°C, the peptide solution was lyophilized using SpeedVac centrifugation and re-suspended with 20 μl 0.1% trifluoroacetic acid (TFA). LC-MS experiments were performed on a Nanoflow HPLC system (Agilent Technologies 1200) connected to a hybrid LTQ-Orbitrap (Thermo Fisher Scientific), equipped with a nanoelectrospray ion source (Proxeon Biosystems). Peptide mixtures were separated by reverse phase chromatography using an Easy-NanoLC analytical column with a two-phase solvent system of 2–40% acetonitrile for 70 min, 40–95% acetonitrile for 5 min and 95% acetonitrile for 5 min in 0.5% acetic acid at a flow rate of 300 μl·min^-1^ and directly electrosprayed into the mass spectrometer. The LTQ-Orbitrap was operated in the data dependent mode to simultaneously measure full scan MS spectra in the Orbitrap and the fifteen most intense ions in the LTQ part by collisionally induced dissociation.

### Enzyme assays of liver tissue

The superoxide dismutase (SOD) activity, the malondialdehyde (MDA) and glutathione (GSH) contents, and the glutathione peroxidase (GSH-Px) and glutathione-S-transferase (GST) activities were measured using commercial assay kits (Jiancheng, Nanjing, China) in accordance with the manufacturer’s instructions.

### Western blot analysis

Protein in the liver was extracted and assayed based on the SDS-PAGE described methods. Samples containing 30 μg proteins were separated by 12.5% SDS-PAGE and transferred onto PVDF membranes. For western blot analysis, the following antibodies were used as the catalog numbers indicate: α-SMA (ab5694, 1:500 dilution), DJ-1 (ab76008, 1:3000 dilution), Bax (sc-23959, 1:200 dilution), Bcl-2 (sc-492, 1:50 dilution), Caspase-3 (sc-7148, 1:100 dilution), Caspase-9 (sc-8355, 1:1000 dilution), AIF (sc-5586, 1:1000 dilution) and GAPDH (sc-25778, 1:1500 dilution).

### TUNEL assay

Apoptotic hepatocytes in the fibrotic liver were evaluated using a TUNEL Apoptosis Detection Kit (Wako Pure Chemical, Osaka, Japan). After four cortical fields were randomly selected from each section, 100 cells were successively counted for each field by an observer. The ratio of the TUNEL-positive cell number to the total cell number is shown.

### Transmission electron microscopy (TEM)

Biopsied liver tissues were cut into pieces (1 × 1 × 5 mm) and fixed with glutaraldehyde in 0.1 M phosphate buffer (pH 7.2) at 4°C for 2 h. After washing in PBS, samples were post-fixed in 1% osmium tetroxide for 1 h at room temperature. The samples were then dehydrated with alcohol, embedded in Quetol 812, sectioned with a diamond knife, stained with 1% uranyl acetate and 1% lead citrate, and examined with a transmission electron microscope (JME-1220; JEOL, Tokyo, Japan) at an acceleration voltage of 80 kV.

### Amino Acids (AA) metabolism analysis

Serum sample preparation for Amino Acids (AA) analysis was performed as previously described [14]. A Waters Acquity system (Waters Corp., Milford, MA, USA) coupled with a triple-quadrupole tandem mass spectrometry was used for the quantification of AA. All data were acquired in centroid mode and processed using MassLynx 4.1 software (Waters Corp., Milford, MA, USA).

### Statistics

All data are presented as mean ± SD. Significant differences were determined by using ANOVA in SPSS 16.0. *P* < 0.05 was considered statistically significant. Protein data analysis was performed with MaxQuant software, supported by Mascot as a database search engine for peptide identification (rat database IPI 3.46) as previously described [15]. Then GO functional annotation analysis of the above different proteins was carried out by GO analysis database (http://www.geneontology.org/), for biological processes (BP) and molecular function (MF). The data of amino acids was analyzed by PCA and PLS-DA. A VIP parameter (Variable Important in Projection, VIP>1) was selected as the cutoff value for searching the most important variables according to the PLS-DA model. In the protein and AA metabolic network, correlation between the differentially expressed proteins and AA biomarkers were analyzed by downloading the pathway data in KEGG Database.

### Materials

*Swertia punicea* was collected from Dali, Yunnan Province, China, and identified by Guang-Xue Liu (Department of Natural Drugs, School of Pharmaceutical Science, Peking University). A voucher specimen (SP-001) was deposited in the herbarium of the Department of Pharmacognosy, School of Pharmaceutical Sciences, Peking University, Beijing. ZYC-1 was extracted and separated from *Swertia punicea* as zheng’s reported method [9]. The purity of the compound ZYC-1 was determined to be more than 98% by high-performance liquid chromatography (HPLC) analysis and the chemical structure of the compound ZYC-1 was shown in the supporting information (S1 Fig).

## Results

### Effects of ZYC-1 on fibrosis markers in serum

**Fig 1:**
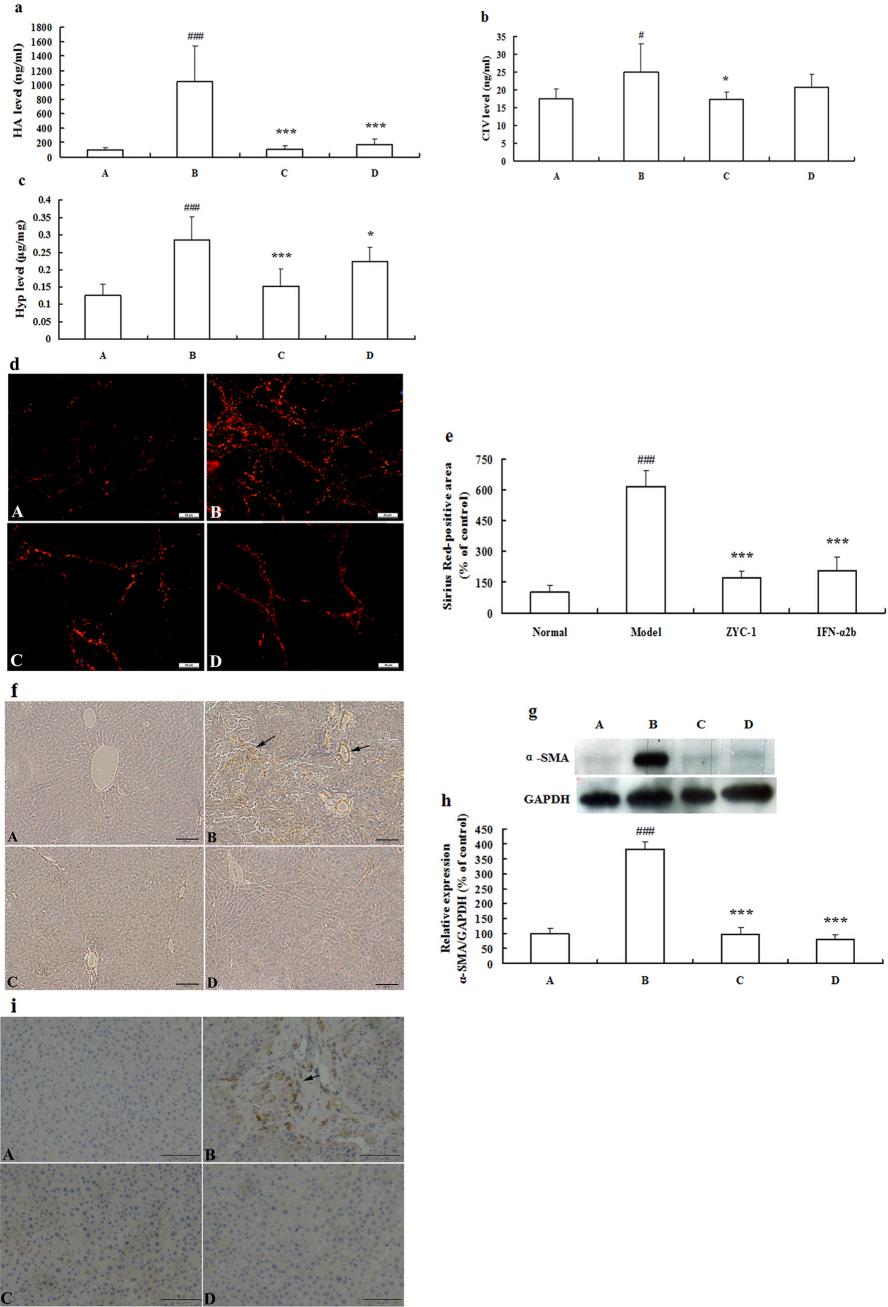
**Effects of ZYC-1 on DMN-induced rat liver fibrosis**: the levels of (a) HA, (b) CIV, and (c) Hyp in the serum of rats. (d) Staining with Sirius Red Bar = 50 μm. (e) Quantitative levels were evaluated by the mean of polarized light intensity using Image Pro Plus 6.0 software in (d). (f) α-SMA expression in liver tissue was analyzed by immunohistochemistry Bar = 100 μm. (g) Protein expression of α-SMA was detected by Western blot, and the gels have been run under the same experimental conditions. Cropped blots are used in (g), and full-length blots are presented in supporting information (S3 Fig). (h) Densitometry analysis is presented as the relative ratio of protein (α-SMA)/GAPDH using Quantity One software (n = 3). (i) TGF-β1 expression in liver tissues was analyzed by immunohistochemistry Bar = 100 μm. (j) Quantitative levels were evaluated by the mean of positive area using Image J software in (i). The data represent the mean ± SD. A: normal group; B: DMN model group; C: ZYC-1 group; D: IFN-α2b group. ^#^*P* < 0.05, ^###^*P* < 0.001 vs. normal group, *P < 0.05, ***P < 0.001 vs. DMN model group.

To explore the effect of ZYC-1 on fibrosis markers, ZYC-1 (5 mg·kg^-1^ body weight) was administrated to DMN induced rats. In model group, DMN treatment significantly increased the levels of the Hyaluronic acid (HA) (*P* < 0.001 vs. normal group), type IV collagen (CIV) (*P* < 0.05 vs. normal group), and Hyp (*P* < 0.001 vs. normal group) (Fig 1C). In the treatment groups, ZYC-1 or Interferon α2b (IFN-α2b) reversed DMN’s effects by decreasing HA level (both *P* < 0.01 vs. model group), and hydroxyproline (Hyp) level (*P* < 0.001 and *P* < 0.05 vs. model group, respectively). In addition, ZYC-1 treatment also decreased the level of CIV (*P* < 0.05 vs. model group) (Fig 1A and 1B).

### Effects of ZYC-1 on histological findings

There was no fibrosis found in the normal group (Fig 1D, A). In the model group, as expected, many bundles grown into the lobules and connected the central and portal areas (*P* < 0.001 vs. normal group) (Fig 1D, B). ZYC-1 treatment attenuated these increases in collagen (*P* < 0.001 vs. model group) (Fig 1D, C), with livers showing only a few tiny, short bundles of collagen. IFN-α2b treatment for 4 weeks also reduced liver fibrosis (*P* < 0.001 vs. model group) (Fig 1D, D). The quantitative levels were evaluated by the mean of polarized light intensity using Image Pro Plus 6.0 software (Fig 1E).

### Effects of ZYC-1 on the expression of α-SMA and TGF-β1

Next, we detected the expression of transforming growth factor beta 1 (TGF-β1) in liver by immunohistochemical staining (Fig 1F and 1I), and α smooth muscle actin (α-SMA) expression by Western blot analysis (Fig 1G). The densitometry analysis is presented as the relative ratio of protein (α-SMA)/GAPDH using Quantity One software (Fig 1H). Compared with normal liver tissue, DMN-treated rats displayed markedly higher expression of α-SMA (*P* < 0.001), and ZYC-1 or IFN-α2b treatment significantly suppressed α-SMA expression (both *P* < 0.001). The variation in TGF-β1 expression between the different groups was similar to α-SMA, although discrepancies were observed (Fig 1I). α-SMA is mainly distributed in the fibrous septal and the inflammatory cell infiltration area after degeneration and necrosis, and TGF-β1 is mainly distributed in the wall of blood sinus, perisinusoidal cells, the non-parenchymal cells of necrosis foci and portal area in the immunohistochemical staining of the DMN-treated group. The quantitative levels of TGF-β1 were evaluated by the mean of positive area using Image J software (Fig 1J).

### Identification of differentially expressed proteins by LC-MS/MS

**Fig 2:**
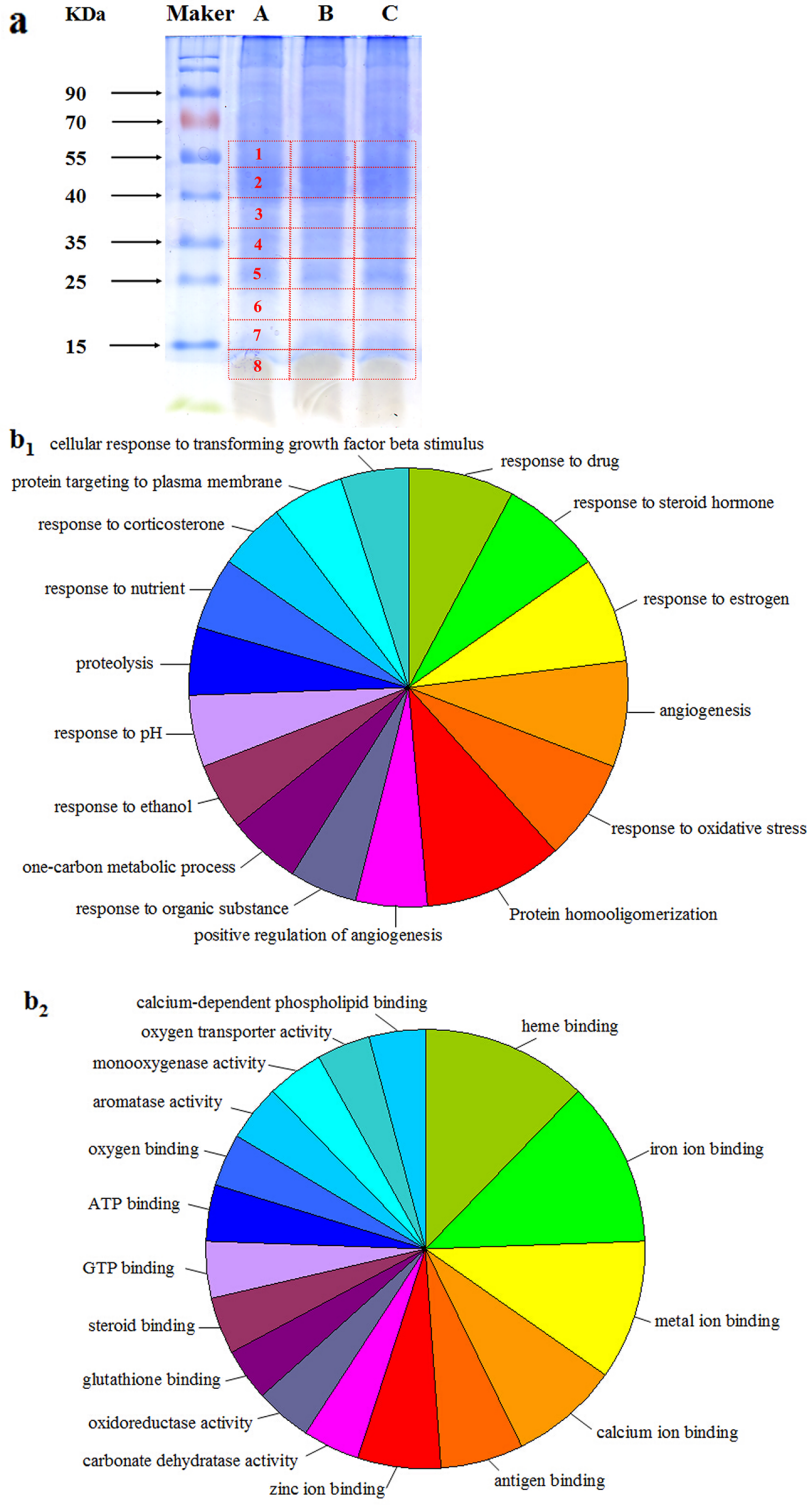
Proteomic analysis of differentially expressed proteins from rat liver in various study groups. Representative 12.5% SDS-PAGE gels (a) showing the total proteins pooled from (A) normal group (n = 3), (B) DMN model group (n = 3) and (C) ZYC-1 group (n = 3). The functional analysis of differentially expressed proteins was carried out by GO analysis database, for the Biological Process (b_1_), Molecular Function (b_2_).

To explore the mechanism involved in ZYC-1 treatment, differently expressed proteins were identified by using LC-MS/MS. Liver proteins were extracted from the normal group (n = 3), the DMN model group (n = 3) and the ZYC-1 treatment group (n = 3) (Fig 2A, From 12.5 kD to 60 kD). In these experiments, a total of 2131 proteins were identified. There were 17 up-regulated proteins (Ratio > 2) and 25 down-regulated proteins (Ration < 0.5) (Table 1).

**Table 1:** The identification of differentially expressed proteins from rat liver in various groups.

Protein IDs	Protein names	Ratio	
		Model versus Normal	ZYC-1 versus Model
G3V913	Heat shock protein beta-1	3.58	0.13
F1LQC1	Acyl-coenzyme A oxidase	0.34	3.28
O88797-2	Disabled homolog 2	2.59	0.40
P01835	Ig kappa chain C region, B allele	3.27	0.27
P01836	Ig kappa chain C region, A allele	6.67	0.13
P01946	Hemoglobin subunit alpha-1/2	4.67	0.36
P02761	Major urinary protein	0.04	69.33
P04176	Phenylalanine-4-hydroxylase	0.42	2.42
P04182	Ornithine aminotransferase	0.34	2.97
P04937-2	Fibronectin	7.03	0.21
P06762	Heme oxygenase 1	6.68	0.14
P08683	Cytochrome P450 2C11	0.12	32.64
P14141	Carbonic anhydrase 3	0.14	13.90
P14668	Annexin A5;Annexin	2.81	0.29
P19225	Cytochrome P450 2C70	3.52	0.30
P20767	Ig lambda-2 chain C region	5.27	0.14
P23680	Serum amyloid P-component	0.13	8.15
P23764	Glutathione peroxidase 3	5.40	0.36
P27139	Carbonic anhydrase 2	2.73	0.39
P27364	3-beta-hydroxysteroid dehydrogenase type 5	0.23	4.34
P29147	D-beta-hydroxybutyrate dehydrogenase	0.31	3.42
P46418	Glutathione S-transferase alpha-5	2.83	0.37
P50430	Arylsulfatase B	4.14	0.28
P52844	Estrogen sulfotransferase	0.03	69.20
P61227	Ras-related protein Rap-2b	2.82	0.33
P68136	Alpha skeletal muscle	5.09	0.22
P70473	Alpha-methylacyl-CoA racemase	0.28	3.99
Q03336	Regucalcin	0.17	5.61
Q07523	Hydroxyacid oxidase 2	0.48	4.23
Q07936	Annexin A2	4.12	0.31
Q0D2L3	Agmatinase	0.37	2.57
Q5FVR2	Thymidine phosphorylase	0.29	10.84
Q641W2	UPF0160 protein MYG1	2.70	0.46
Q64648	Cytochrome P450 2C12	7.86	0.12
Q68G41	Enoyl-CoA delta isomerase 1	0.45	2.18
Q6AYC4	Macrophage-capping protein	4.65	0.30
Q6IMY6	Lysosomal acid lipase/cholesteryl ester hydrolase	3.43	0.40
Q6P6T6	Cathepsin D	2.98	0.38
Q8R491	EH domain-containing protein 3	0.39	2.65
Q8R5M5	2-amino-3-carboxymuconate-6-semialdehyde decarboxylase	3.29	0.31
Q921A4	Cytoglobin	6.54	0.24
Q9R0J8	Legumain	3.43	0.31

Our results also showed that these 42 proteins were mainly involved in several biological processes (BP) (Fig 2B_1_) and molecular functions (MF) (Fig 2B_2_). The detailed MF or BP information of these proteins was showed in Supporting information (S1 Table). Among these 42 proteins, acyl-coenzyme A oxidase, glutathione peroxidase 3 and Valley glutathione S-transferase enzyme α-5 were related to oxidative stress; heat shock protein beta-1 was linked to apoptosis by preventing activation of caspases; hydroxy acid oxidase 2, benzene homoalanin-4-hydroxylase and ornithine aminotransferase were associated with amino acid metabolism. According to the results of proteomics analysis, ZYC-1 may act through the oxidative stress pathway, mitochondrial-mediated apoptotic pathway and regulate the balance of amino acids to exert its anti-fibrosis action.

### ZYC-1 attenuates DMN-induced oxidative stress

**Fig 3:**
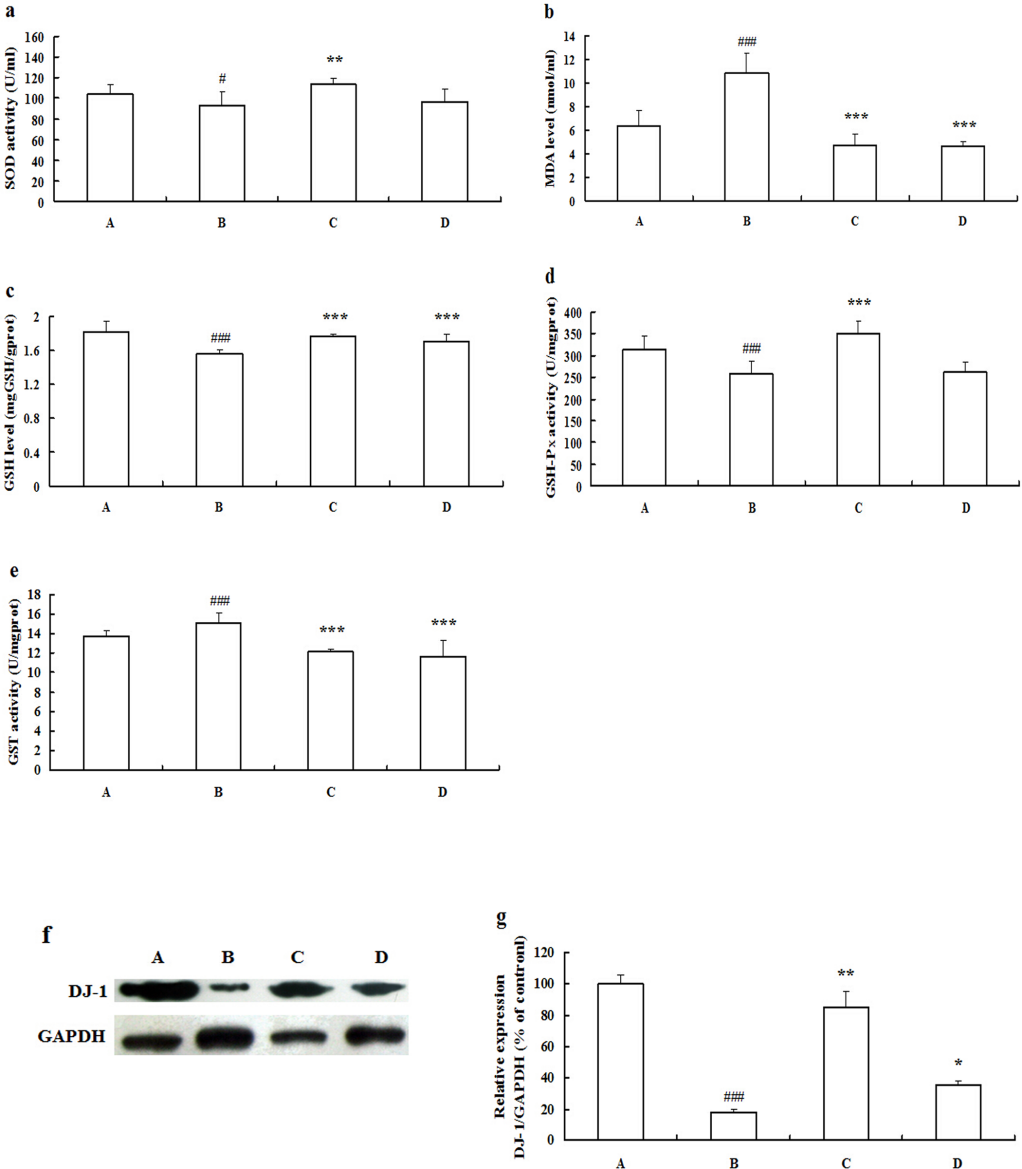
Effects of ZYC-1 on oxidative stress in the liver of rats treated with DMN. (a) SOD activities, (b) MDA levels, (c) GSH levels, (d) GSH-P_X_ activities and (e) GST activities. (f) The expression level of DJ-1 protein was detected by Western blot, and the gels have been run under the same experimental conditions. Cropped blots are used in (f), and full-length blots are presented in supporting information (S4 Fig). Densitometry analysis is presented as the relative ratio of protein (DJ-1)/GAPDH using Quantity One software. The data represent the mean ± SD. A: normal group; B: DMN model group; C: ZYC-1 treatment group; D: IFN-α2b treatment group. ^#^*P* < 0.05, ^##^*P* < 0.01, ^###^*P* < 0.001 vs. normal group, *P < 0.05, **P < 0.01, ***P < 0.001 vs. DMN model group.

In the Fig 3A and 3B, about 10% fall in the superoxide dismutase (SOD) activity (*P* < 0.05 vs. normal group) and about 1.5-fold increase in malondialdehyde (MDA) (*P* < 0.001 vs. normal group) formation occurred following DMN treatment. Treatment with ZYC-1 following DMN treatment significantly promoted the SOD activity and prevented the formation of MDA (*P* < 0.05 and *P* < 0.001 vs. model group, respectively). Meanwhile, IFN-α2b treatment also decreased the MDA contents (*P* < 0.001 vs. model group). The glutathione (GSH) level and the glutathione peroxidase (GSH-Px) activity decreased markedly (both *P* < 0.001 vs. normal group), while glutathione-S-transferase (GST) activity increased significantly in model rats (*P* < 0.001 vs. normal group) (Fig 3C, 3D and 3E). In comparison with the DMN model group, the degree of fibrosis was evidently milder with higher levels of GSH, higher GSH-Px activity and lower GST activity in rats treated by ZYC-1 (all *P* < 0.001 vs. model group). Meanwhile, IFN-α2b treatment also increased GSH levels and decreased GST activity (both *P* < 0.001 vs. model group). The expression level of DJ-1 protein in DMN model rats was significantly decreased (*P* < 0.001 vs. normal group). The administration of ZYC-1 or IFN-α2b was shown to alleviate these changes (both *P* < 0.001 vs. model group) (Fig 3F and 3G).

### ZYC-1 attenuated the expression level of DMN-induced apoptosis-related proteins

**Fig 4:**
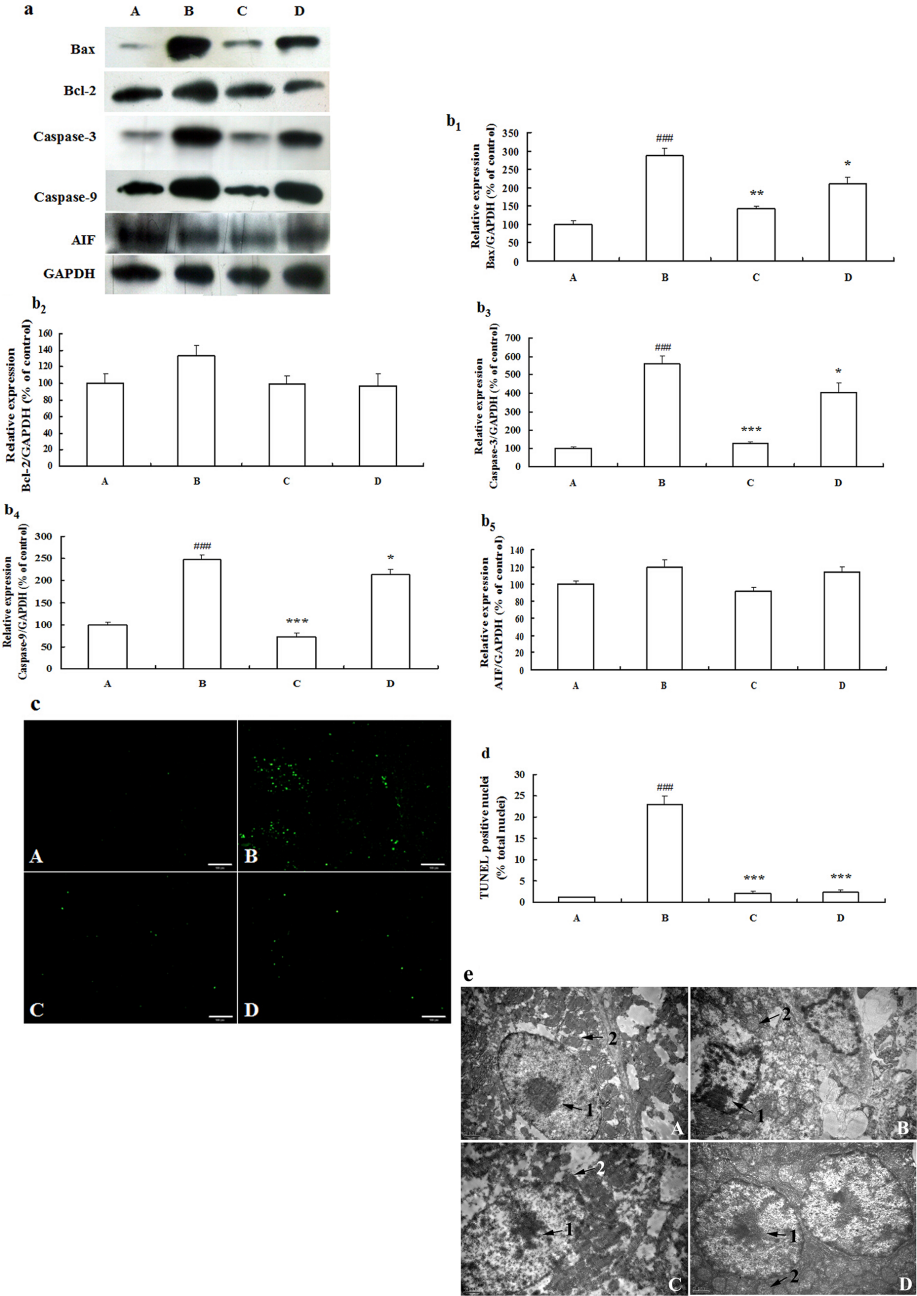
Effects of ZYC-1 on apoptotic index in the liver of rats treated with DMN. (a) Protein expression levels of Bax, Bcl-2, Caspase-3, Caspase-9 and AIF was detected by Western blot, and the gels have been run under the same experimental conditions. Cropped blots are used in (a), and full-length blots are presented in supporting information (S5 Fig). (b_1-5_) Densitometry analysis is presented as the relative ratio of protein (Bax, Bcl-2, Caspase-3, Caspase-9 and AIF)/GAPDH using Quantity One software. (c) The TUNEL assay was observed under fluorescence microscopy. Bar = 100 μm. (d) Statistic analysis of TUNEL was evaluated by fluorescence intensity using Image Pro Plus 6.0 software. (e) TEM observation of liver apoptosis. The morphological alternations of nuclei (1) and mitochondria (2) (as arrows showed) indicated in TEM images. A: normal group; B: DMN model group; C: ZYC-1 treatment group; D: IFN-α2b treatment group. Bar = 1 μm. Data represent the mean *±* S.D. ^###^*P* < 0.001 vs. normal group, *P < 0.05, **P < 0.01, ***P < 0.001 vs. DMN model group.

In Fig 4A, the level of Bax protein increased following DMN treatment (*P* < 0.001). ZYC-1 or IFN-α2b decreased the expression level of Bax (*P* < 0.01 and *P* < 0.05). However, no significant change in the Bcl-2 level was observed among different groups. There were obvious increases in the caspase-3 and caspase-9 expression levels in the DMN model group (both *P* < 0.001). Treatment with ZYC-1 (both *P* < 0.001) or IFN-α2b (both *P* < 0.05) prevented the DMN-related increase of caspase-3 and caspase-9 levels. No significant change in the level of AIF was found among different groups. The densitometry analysis is presented as the relative ratio of protein (Bax, Bcl-2, Caspase-3, Caspase-9 and AIF)/GAPDH using Quantity One software (Fig 4B_1-5_).

### ZYC-1 attenuated the morphological changes of apoptosis in DMN-induced liver fibrosis rats

Few TUNEL-positive cells were observed in normal rats (Fig 4C). By contrast, the ratio of number of the TUNEL-positive cells / total number of the cells clearly increased in DMN-induced liver fibrosis rats (41.67% ± 8.39, *P* < 0.001). ZYC-1 or IFN-α2b treatment could evidently decreased the ratio of number of the TUNEL-positive cells / total number of the cells in DMN model rats (14.13 ± 4.26 and 20.65 ± 6.78, both *P* < 0.001). The TEM results further revealed the status of nuclei and mitochondria in different groups, showed in Fig 4D. Taken together, compared with normal rats, a condensed nucleus with chromatin condensation and margination, and mitochondria cristae disappearance were observed in DMN-induced hepatocytes. The cellular morphology indicated that ZYC-1 or IFN-α2b treatment could markedly decrease nuclear and mitochondrial injury. Moreover, cells under treatment exhibited approximate oval appearance and intact hepatocytes membrane, and the condensation of chromatin within the nucleus is improved significantly.

### Content analysis of Amino acid (AA)

The concentrations of His, Phe and Arg were found to be up-regulated in DMN-induced rat serum (*P* < 0.05, *P* < 0.001 and *P* < 0.05, respectively). After ZYC-1 treatment, the concentrations of Phe and Arg were significantly lower than those in the DMN-induced model groups (both *P* < 0.05). Meanwhile, the concentrations of Leu/Ile and Thr were down-regulated in DMN-induced rat serum (*P* < 0.01 and *P* < 0.001, respectively). On the other hand, after ZYC-1 treatment, the concentrations of Leu/Ile and Thr were higher than that of DMN-induced model groups (both *P* < 0.01). After IFN-α2b treatment, the concentrations of His, Phe and Thr were higher than that of DMN-induced model groups (*P* < 0.05, *P* < 0.01 and *P* < 0.01, respectively) (Table 2).

**Table 2:** Contents of AAs (μg/mL) measured using UPLC-MS in the rat serum in various groups.

No.	AA	Normal (n = 6)	Model (n = 6)	ZYC-1 (n = 3)	IFN-α2b (n = 6)
1	Glu	5.01 ± 3.50	7.21 ± 4.30	10.21 ± 6.69	5.19 ± 3.65
2	His	25.76 ± 4.75	34.77 ± 8.06^#^	29.25 ± 5.47	19.65 ± 8.88*
3	Phe	21.87 ± 1.57	44.13 ± 8.55^###^	26.63 ± 3.40*	26.44 ± 4.90**
4	Trp	34.11 ± 5.26	38.12 ± 14.02	37.36 ± 3.08	32.97 ± 7.79
5	Met	16.72 ± 2.63	14.59 ± 2.76	21.06 ± 3.00*	11.57 ± 7.58
6	Leu/Ile	50.94 ± 8.04	38.28 ± 5.27^##^	56.38 ± 8.96**	34.50 ± 9.93
7	Val	32.89 ± 5.85	27.38 ± 4.98	41.86 ± 2.55**	24.27 ± 7.61
8	Pro	44.79 ± 11.03	41.87 ± 12.22	59.15 ± 10.53	35.02 ± 5.14
9	Thr	6.94 ± 0.85	3.86 ± 1.18^###^	11.17 ± 2.56**	8.35 ± 0.40**
10	Ala	77.39 ± 47.76	63.79 ± 47.59	122.34 ± 17.77	32.94 ± 38.67
11	Gln	19.98 ± 1.71	19.53 ± 3.77	21.52 ± 2.47	35.79 ± 20.04
12	Asp	5.58 ± 4.90	6.24 ± 2.58	11.45 ± 1.92*	3.76 ± 1.77
13	Ser	24.29 ± 4.26	19.15 ± 6.56	29.31 ± 2.43*	19.73 ± 8.80
14	Lys	118.18 ± 12.33	121.47 ± 22.48	99.10 ± 10.46	100.24 ± 24.40
15	Arg	28.56 ± 13.60	49.17 ± 15.19^#^	21.85 ± 10.27*	35.22 ± 9.50

The data represent the mean ± SD.

^#^*P* < 0.05, ^##^*P* < 0.01, ^###^*P* < 0.001 vs. normal group, *P < 0.05, **P < 0.01 vs. DMN model group.

### Multivariate statistics analysis of AA

**Fig 5:**
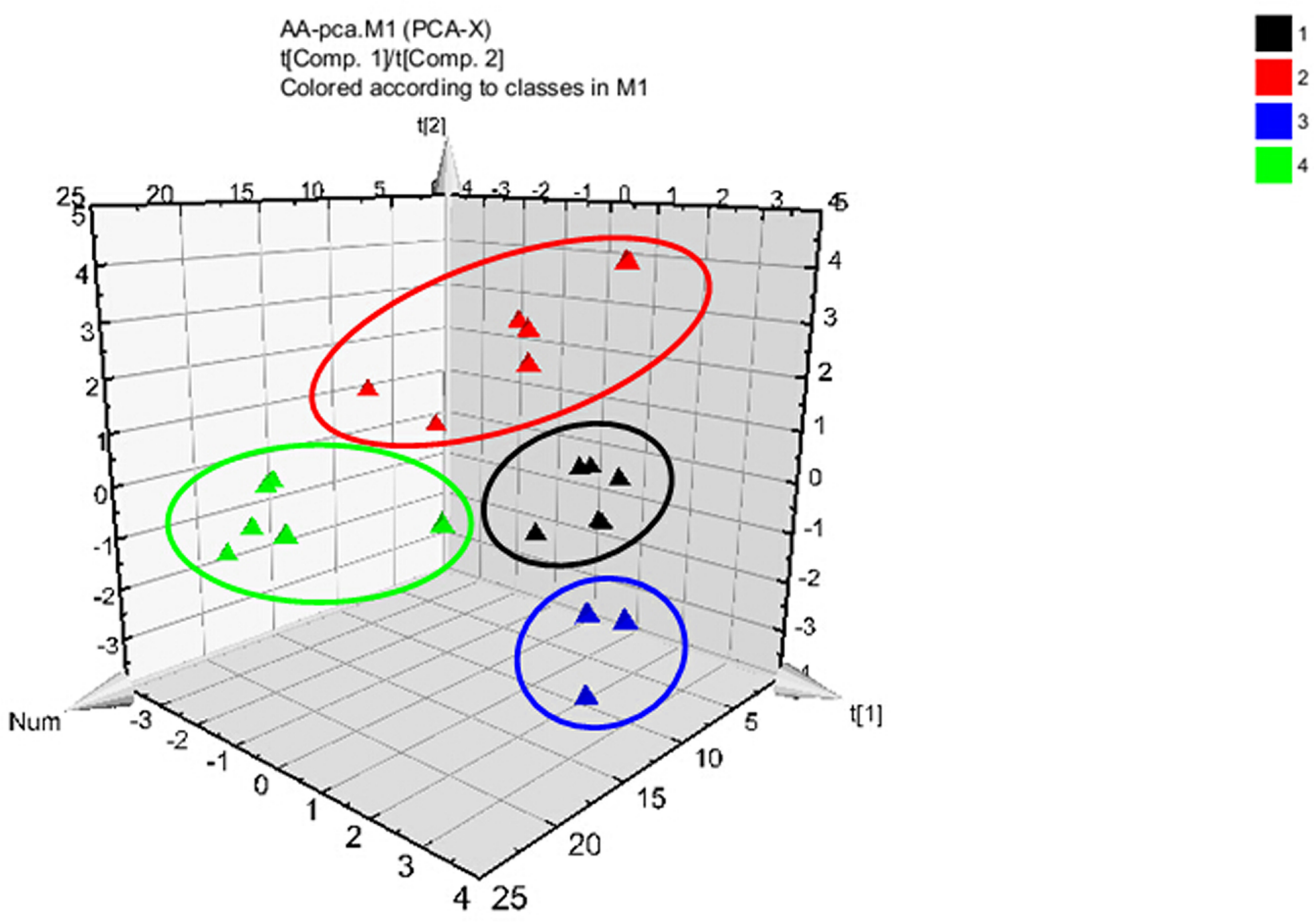
The PCA analysis of amino acids among normal groups (black), DMN model groups (red), ZYC-1 treatment groups (blue) and IFN-α2b treatment groups (green). t[1] and t[2] are the first two eigenvectors of original data.

After the data of AA standardization, 15 AA variables were used for Principle components analysis (PCA), respectively. The first two principal components (PC1 and PC2) could explain 52.5% of the AA variability, and the plots of first two PC scores were shown in Fig 5. We found that the metabolic pattern was different among the normal, DMN model, ZYC-1 and IFN-α2b treatment groups. Then, a Partial least squares discriminate analysis (PLS-DA) model was developed to discriminate among the four groups and identify the potential AA biomarkers. The R^2^X, R^2^Y and Q^2^Y derived from the 15 AA variables were 90.4, 67.3 and 52.9% respectively. Components that played important roles in the classification were picked out according to the parameter of VIP (Variable Importance in Projection) value.

The correlation analysis was conducted using Pearson correlation analysis to probe the relationship between the concentrations of the AA biomarkers (variable importance, VIP > 1) and the levels of HA, CIV and Hyp. As an example, the results showed the characteristic inverse correlation between the level of Thr and the level of HA (R = -0.663, *P* < 0.01) or CIV (R = -0.556, *P* < 0.05), as well as the typical positive correlation between the level of Phe and the level of HA (R = 0.894, *P* < 0.001), CIV (R = 0.787, *P* < 0.001) or Hyp (R = 0.807, *P* < 0.001). It would appear that AA had a significant correlation with DMN-induced liver fibrosis, and the serum AA levels and the metabolic profiles indicated that ZYC-1 possibly improved amino acid metabolism, which was one purpose of treatment against liver fibrosis.

Furthermore, the Kyoto Encyclopedia of Genes and Genomes (KEGG) pathway analysis between differentially expressed proteins and AA biomarkers confirmed that differently expressed proteins from rat liver in various groups were significantly correlated with AA biomarkers in the same pathway (Table 3). For example, Phenylalanine-4-hydroxylase, which is the key protein of the pathway of Protein digestion and absorption, was correlated with AA biomarkers, such as Lys, Asp, Arg, Gln, Ser, Met, Trp, Phe, Leu, His, Pro, Val and Thr. Also, in the arginine and proline metabolism pathway, there was close relation between differently expressed proteins, such as Ornithine aminotransferase and Agmatinase and corresponding AA biomarkers.

**Table 3:** Differently expressed proteins from rat liver in various groups related to AA biomarkers in the same pathway using KEGG pathway analysis.

Gene	Entrez	Protein	EC	Pathway name consulted from KEGG	Relative AA biomarkers
Pah	24616	Phenylalanine-4-hydroxylase	1.14.16.1	Protein digestion and absorption	Lys; Asp; Arg; Gln; Ser; Met; Trp; Phe; Leu; His; Pro; Val; Thr
				PhenylAla, tyrosine and tryptophan biosynthesis	Trp; Phe
				PhenylAla metabolism	Phe
Oat	64313	Ornithine aminotransferase	2.6.1.13	Biosynthesis of antibiotics	Lys; Asp; Arg; Ser; Phe; Pro; Val; Thr; Glu
				Arginine and proline metabolism	Arg; Pro
Hmox1	24451	Heme oxygenase 1	1.14.99.3	Mineral absorption	Gln; Ser; Met; Trp; Phe; Leu; Pro; Val; Thr
				Porphyrin and chlorophyll metabolism	Thr
Ca2	54231	Carbonic anhydrase 2	4.2.1.1	Nitrogen metabolism	Gln
Rgn	25106	Regucalcin	3.1.1.17	Biosynthesis of antibiotics	Lys; Asp; Arg; Ser; Phe; Pro; Val; Thr; Glu
				Microbial metabolism in diverse environments	Lys; Asp; Gln; Ser; Thr; Glu
				Carbon metabolism	Asp; Ser; Glu
				Pentose phosphate pathway	Glu
Hao2	84029	Hydroxyacid oxidase 2	1.1.3.15	Biosynthesis of antibiotics	Lys; Asp; Arg; Ser; Phe; Pro; Val; Thr; Glu
				Microbial metabolism in diverse environments	Lys; Asp; Gln; Ser; Thr; Glu
				Carbon metabolism	Asp; Ser; Glu
				Glyoxylate and dicarboxylate metabolism	Gln; Ser
Agmat	298607	Agmatinase	3.5.3.11	Arginine and proline metabolism	Arg; Pro
Tymp	315219	Thymidine phosphorylase	2.4.2.4	Pyrimidine metabolism	Gln
Cyp2c12	25011	Cytochrome P450 2C12	1.14.14.1	Serotonergic synapse	Trp
Ctsd	171293	Cathepsin D	3.4.23.5	Sphingolipid signaling pathway	Ser
Acmsd	171385	2-amino-3-carboxymuconate-6-semialdehyde decarboxylase	4.1.1.45	Tryptophan metabolism	Trp

## Discussion and conclusions

Liver fibrosis can end up with the development of hepatic cirrhosis and hepatocellular carcinoma. To date, no drugs have been reported to inhibit liver fibrosis completely. ZYC-1, one of the main active ingredients of *Swertia punicea* (Gentianaceae), possesses hepatoprotective activity [9]. However, the hepatoprotective effect of ZYC-1 is still needs further confirmation and the hepatoprotective mechanisms are not fully understood. We therefore used a DMN-induced rat liver fibrosis model to further assess its anti-liver fibrosis effects and investigated its possible mechanism.

The level change of CIV, PCIII, HA, LN and Hyp is commonly used to study the mechanisms of liver fibrosis and the therapeutic effects of drugs [16]. The present study demonstrated that ZYC-1 prevented the development of liver fibrosis as confirmed by quantitative measurement of serum levels of HA and CIV. ZYC-1 also reduced Hyp levels, which could reduce the availability of Hyp for collagen synthesis, as indicated by Picro Sirius red staining. However, no significant difference was observed in the levels of LN and PCIII (data not showed).

Activated HSCs are the main source of extracellular matrix in fibrotic liver, and α-SMA and TGF-β1 are known as a specific marker of HSCs activation. Their molecular mechanism relates with the process of epithelial to mesenchymal transition (EMT) [17, 18]. Our studies indicated that considerable α-SMA and TGF-β1 expressions were observed in the rat liver in the model group compared to the control group, while their expression levels were significantly reduced by ZYC-1, suggesting that the anti-fibrosis effects of ZYC-1 may be related to the inhibition of TGF-β1 and TGF-β1-induced EMT.

Proteomics is known to be a large-scale comprehensive study of proteins [19], especially, it is a powerful tool for studying changes in protein expression and identifying biomarkers for pathogenic processes [20]. The proteomics analysis of liver fibrotic animals and cells could provide substantial information for a better understanding of liver fibrogenesis, identify potential diagnostic markers, and discover therapeutic target candidates [21, 22]. In the present study, we examined several indicators of liver fibrosis to confirm the anti-liver fibrosis activities of ZYC-1. We then used Liquid Chromatography coupled with a tandem Mass Spectrometry (LC-MS/MS) proteomics approach to separate and identify 42 differentially expressed proteins in the DMN model versus normal, and in ZYC-1 versus DMN model in rat liver. According to the results of proteomics analysis, ZYC-1 may act through the oxidative stress pathway, mitochondrial-mediated apoptotic pathway and regulate the balance of amino acids to exert its anti-fibrosis action.

Apoptosis might be attributed to the presence of oxygen-derived free radicals and other ROS, and normal cells are well equipped with a series of antioxidant protective systems in order to neutralize the damages of ROS to prevent apoptosis [23]. Branched-chain amino acids (BCAA) treatment increases the ratio of reduced albumin, which in turn down regulate oxidative stress by modulating the redox state of albumin in patients with cirrhosis [24]. Meanwhile, BCAA supplementation attenuates hepatocyte apoptosis in rats with liver damage via the control of Bax localization [25]. Further study of the effects of ZYC-1 on oxidative stress, apoptosis and amino acid imbalance in DMN rat model were shown as follows.

Oxidative stress plays an important role in the formation of liver fibrosis via intensive the stellate cell activation and collagen synthesis [26]. SOD, an antioxidant enzyme for oxidative stress protection and ROS detoxification, and also MDA, the end product of lipid peroxidation, have been shown to activate the collagen producing stellate cells [27]. DJ-1 is a redox-sensitive protein that scavenges ROS by increasing glutathione synthesis [28]. DJ-1 expression is significantly altered in hepatocellular carcinoma (HCC), which suggests that DJ-1 may be a candidate prognostic biomarker of HCC [29]. In our study, the beneficial effect was shown by reversal of SOD, MDA, GSH, GSH-Px, GST and DJ-1 in the liver tissues of rats treated with ZYC-1, and the results confirmed the compound’s antioxidant effect against DMN-induced oxidative stress. The effects of ZYC-1 on the SOD, GSH, GST and other activities, whether by regulating DJ-1 expression to implement the above changes, still remains to be further studied in the future.

Apoptosis has an important role in the pathogenesis of liver fibrosis [30]. Aiming at further identifying whether ZYC-1 functions through the apoptotic pathway, we determined the expression levels of certain key apoptosis-related proteins in the Bcl-2 family and mitochondria-mediated signaling pathway. The Bcl-2 family consists of two subfamilies: anti-apoptotic factors such as Bcl-2 and pro-apoptotic factors such as Bax [31]. Bcl-2, located in the outer mitochondria membrane, has been reported to block apoptosis by inhibiting the release of apoptosis inducing factors from mitochondria, or by blocking the activation of effector caspases [32]. In this study, Bax was increased but Bcl-2 showed no significant changes after DMN treatment. ZYC-1 treatment decreased Bax expression levels. These results suggested that ZYC-1 protects the liver partly through the regulation the Bcl-2/Bax levels. ZYC-1 also down-regulated DMN-induced apoptosis by activation of the caspase cascade. In addition, AIF showed no significant changes among the different groups. The anti-apoptosis effects of ZYC-1 may not be realized through the caspase-independent mitochondria mediated cell death signaling pathway.

In DMN model group, TUNEL staining indicated the production of DNA fragments. The necrosis and apoptosis of hepatocytes attributes to cell death in the centrilobular area. Condensed and marginal nucleus, a hallmark of apoptosis, was observed in the TEM image after DMN exposure. However, treatment with ZYC-1 protected cells from nuclear loss and condensation as well as inhibiting DNA fragmentation, which was beneficial in maintaining the normal cellular structure and function against DMN-induced hepatotoxicity.

Amino acid imbalance has been well studied in patients with acute liver failure or with liver fibrosis [33]. The serum ratio of BCAA comprising valine (Val), leucine (Leu) and isoleucine (Ile) and aromatic amino acids (AAA) comprising tryptophan (Trp) and phenylalanine (Phe) is known as Fisher’s ratio, and it has been reported to be decreased in patients with liver fibrosis [34]. The value of BCAA/AAA in the normal groups was 1.46, whereas the corresponding value in the model groups was 0.79. Following ZYC-1 treatment, the BCAA/AAA ration in the ZYC-1 treatment groups was 1.54. This result demonstrated the promise of combining analytical methods with advanced statistical methods based on metabolomics, which not only explained the mechanism of ZYC-1 treatment, but also elucidated the diagnostic and prognostic value in various liver diseases. In addition, in the same pathway, such as Protein digestion and absorption, PhenylAla, tyrosine and tryptophan biosynthesis, and PhenylAla metabolism, the differentially expressed proteins identified by LC-MS/MS significantly correlated with the AA biomarkers. In conclusion, the above experiments confirmed the results of proteomics analysis.

In conclusion, ZYC-1 can effectively inhibit liver fibrosis. In order to elucidate the anti-fibrotic mechanism of ZYC-1, we demonstrated that ZYC-1 inhibited the activation of HSCs via down-regulation of α-SMA and TGF-β1. The decrease of Hyp level might inhibit the collagen synthesis. Based on proteomics results and further verification from three different aspects, ZYC-1 may act through the oxidative stress pathway, the apoptosis pathway, and regulation of the balance of amino acids to exert its anti-liver fibrosis effects. In the future course of the study, following the results of proteomics analysis, we will further investigate other differentially proteins such as calcium-dependent phospholipid binding protein etc.

Our study presented the first elucidated mechanisms of xanthone on liver fibrosis *in vivo*, and warrants further studies of ZYC-1 as a potential treatment in a clinical setting.

## Supporting information

S1 Fig The structure of the compound 1,7-dihydroxy-3,4,8-trimethoxyxanthone (ZYC-1) isolated from *Swertia punicea Hemsl*. (TIF)

S2 Fig The flowchart of animal experiment. (TIF)

S3 Fig Protein expression of α-SMA was detected by Western blot. (TIF)

S4 Fig The expression level of DJ-1 protein was detected by Western blot. (TIF)

S5 Fig Protein expression of Bax, Bcl-2, Caspase-3, Caspase-9 and AIF was detected by Western blot. (TIF)

S1 Table The detailed MF or BP information of the differentially expressed proteins. (CSV)
