# Supplementary figures and images for: Antioxidant, antiapoptotic and amino acid balance regulating activities of 1,7-dihydroxy-3,4,8-trimethoxyxanthone against dimethylnitrosamine-induced liver fibrosis
Source: PLoS One. 2017 Dec 12;12(12):e0189344. doi: 10.1371/journal.pone.0189344 (PMC5726633; doi:10.1371/journal.pone.0189344)

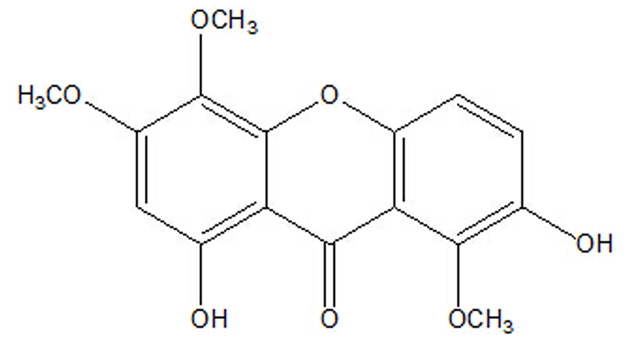

Supplement: S1 Fig — (TIF) [file pone.0189344.s001.tif]

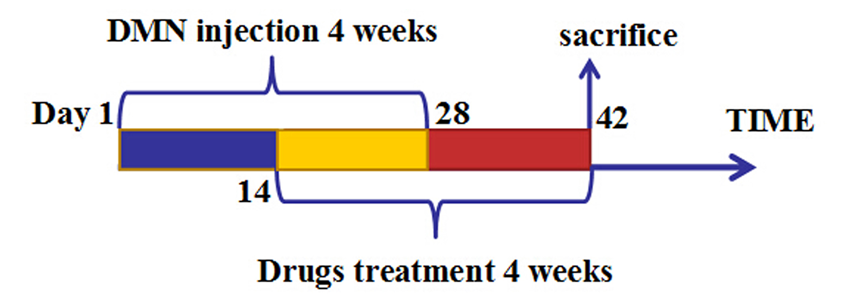

Supplement: S2 Fig — (TIF) [file pone.0189344.s002.tif]

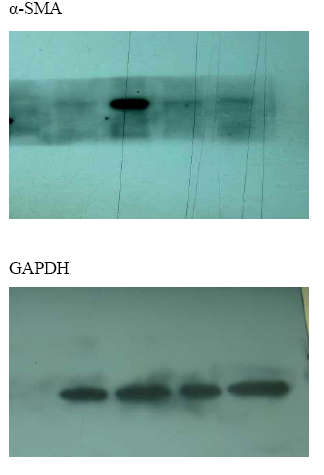

Supplement: S3 Fig — (TIF) [file pone.0189344.s003.tif]

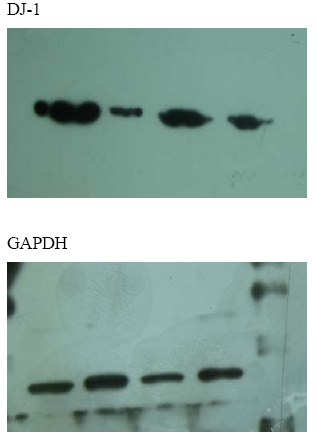

Supplement: S4 Fig — (TIF) [file pone.0189344.s004.tif]

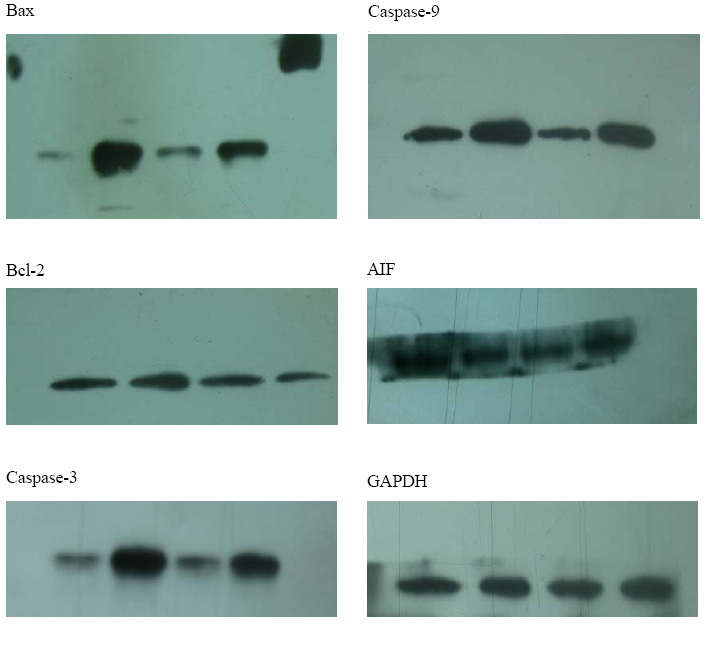

Supplement: S5 Fig — (TIF) [file pone.0189344.s005.tif]
